# Differentiating Choroidal Melanomas and Nevi Using a Self-Supervised Deep Learning Model Applied to Clinical Fundoscopy Images

**DOI:** 10.1016/j.xops.2024.100647

**Published:** 2024-11-08

**Authors:** Max Jackson, Helen Kalirai, Rumana N. Hussain, Heinrich Heimann, Yalin Zheng, Sarah E. Coupland

**Affiliations:** 1 Liverpool Ocular Oncology Research Group, Department of Eye and Vision Science, Institute of Life Course and Medical Sciences (ILCaMS), University of Liverpool, Liverpool, United Kingdom; 2 Liverpool Ocular Oncology Centre, Liverpool University Hospitals Foundation Trust (LUHFT), Liverpool, United Kingdom; 3 Liverpool Centre for Cardiovascular Science at University of Liverpool, Liverpool John Moores University and Liverpool Heart and Chest Hospital, Liverpool, United Kingdom; 4 Department of Eye and Vision Science, ILCaMS, Liverpool, United Kingdom; 5 Liverpool Clinical Laboratories, LUHFT, Liverpool, United Kingdom

**Keywords:** Choroidal melanoma, Deep learning, Foundation models, Fundoscopy, Nevi

## Abstract

**Purpose:**

Testing the validity of a self-supervised deep learning (DL) model, RETFound, for use on posterior uveal (choroidal) melanoma (UM) and nevus differentiation.

**Design:**

Case-control study.

**Subjects:**

Ultrawidefield fundoscopy images, both color and autofluorescence, were used for this study, obtained from 4255 patients seen at the Liverpool Ocular Oncology Center between 1995 and 2020.

**Methods:**

After excluding poor-quality images, a total of 18 510 UM, 8671 nevi, and 1192 healthy eye images were analyzed. RETFound, a self-supervised DL model for fundus images, was fine-tuned initially for binary classification of UM versus nevi and then retuned for tertiary classification including the healthy eyes.

**Main Outcome Measures:**

The performance metrics used to evaluate the model were: area under the receiver operating characteristic curve (AUROC), accuracy, specificity, sensitivity, F1-score, and Matthew’s correlation coefficient.

**Results:**

For the binary classification task, the model achieved an accuracy of 0.83 and an AUROC of 0.90 demonstrating good performance for UM versus nevi differentiation. Similarly, for the tertiary classification task, the model showed a mean accuracy of 0.82 and an AUROC of 0.92.

**Conclusions:**

Our findings demonstrate the feasibility of using a self-supervised DL model for differentiation between UM and nevi with high accuracy, in a large cohort with imbalances between images derived from a single center. Validation studies on similarly sized external cohorts are planned to test our model’s potential, considering variation of images of choroidal melanoma and nevi in the clinical setting.

**Financial Disclosure(s):**

Proprietary or commercial disclosure may be found in the Footnotes and Disclosures at the end of this article.

Uveal melanoma (UM) is a rare eye cancer affecting around 6 individuals per million per year.^1^ It is the most common primary intraocular malignancy in adults and arises in the choroid, iris, and ciliary body,^2^ with the posterior (choroidal) melanomas being the most frequent.^3^ Uveal melanoma metastasis occurs in approximately 50% of cases, usually spreading to the liver.^4^ Once metastasis occurs, patient prognosis is generally poor with a median survival time of <1 year^5^ because curative treatment options are limited at present. Hence, there is an urgent medical need to improve earlier detection and treatment of UM, both within the eye and of the metastases in the liver.

Most choroidal melanomas are diagnosed clinically (i.e., through a variety of nonionizing imaging techniques).^6^ The 3 most common techniques used to establish the diagnosis of melanoma include color fundus imaging (Fig 1), ultrasonography, and OCT.^6^ Each method provides disparate but cumulative information, which provides sufficient evidence to establish the diagnosis of melanoma. Despite this, differentiating between UM and nevi can be difficult.

**Figure 1:**
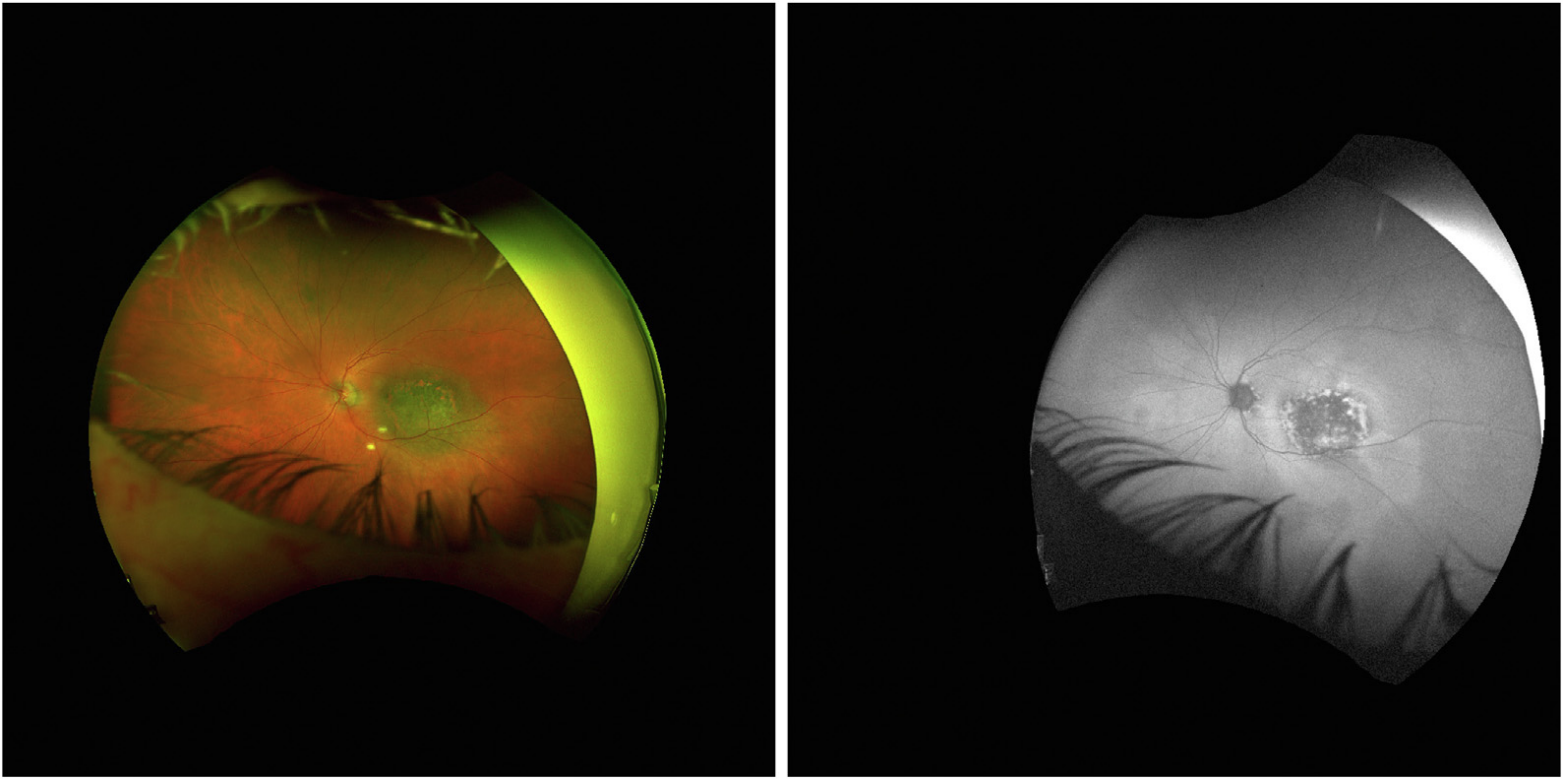
Example of Optos fundus images from the Liverpool Ocular Oncology Center with the left being color fundus and right autofluorescence.

Nevi are “freckles” that can arise in the iris or choroid through the clustering and proliferation of normal melanocytes without any evidence of cellular atypia. Nevi may share some common clinical features of small UM, their malignant counterpart. Some classic features that point toward a diagnosis of UM are as follows: presence of orange pigment (lipofuscin), thickness (>2 mm), largest basal diameter (LBD) (>5 mm), subretinal fluid, intertumoral vascularity, and lack of drusen.^7^ If the lesion is atypical in appearance, an intraocular biopsy can be taken and analyzed in the pathology laboratory to determine the nature of the lesion.^8^ Correct clinical diagnosis by the ophthalmic oncologist at specialist referral centers for the differentiation between nevi and UM is very high; however, many referrals to ocular oncology specialist centers come from local hospitals or opticians, where up to 23% of UM are misdiagnosed.^9^

Artificial intelligence (AI) (also known as “augmented” intelligence) is being widely applied in health care, including in ophthalmology, for both fundus and OCT images. It has shown very promising results for the earlier diagnosis and detection of diabetic retinopathy and age-related macular degeneration.^10^ However, the use of AI for UM images is limited to date. In this study, a self-supervised foundation deep learning (DL) model, RETFound,^10^ was applied to determine if it could aid in the differentiation between choroidal melanoma and nevi.

## Methods

This study conformed to the principles of the Declaration of Helsinki and all procedures and methods relating to the data used were approved by the Health Research Authority under the REC Ref 20-LO-1126.

### Patients and Equipment

Pseudo-anonymized data were obtained through the Liverpool Ocular Oncology Centre with 3942 patients’ fundus images obtained from 1995 to 2020. Images were taken on an Optos ultrawidefield model P200 camera.^11^

### Images: Exclusion Criteria

During the preprocessing of the images used in this study, specific criteria were set to exclude images deemed to be of poor quality or unsuitable. Images were excluded if (1) they were blurry or of low resolution; (2) they had the presence of artifacts, such as eyelashes protruding across the whole image; or (3) most of the eye was blocked by camera distortion. Other excluding factors included the following: eyes with vitreous hemorrhage; a lesion that was over 50% out of view; dense cataract; no visible vascular structures; and any nevus that had subsequently transformed into a melanoma. Images that were post treatment (and therefore usually contained some scarring) were still included in this study. By applying class activation maps to visualize what the model uses for classification, the white scarring did not show up in any significant way. This study concentrated on choroidal melanomas because (1) iris melanomas did not have any fundus images because of the tumor location and (2) ciliary body melanomas rarely had a comparative nevus group.

The remaining 27 181 good-quality images were classified as either posterior (choroidal) UM or nevi based on the clinical diagnosis. The diagnosis of the UM and nevus patients was undertaken by 2 experienced ocular oncologists involving direct patient examination, Optos^11^ and autofluorescence imaging, OCT scans, and ultrasonography. The diagnosis was based on the known clinical features pertaining to tumor dimensions, orange pigment, subretinal fluid, and ultrasound features of reflectivity and Doppler phenomenon.^12^ Additional information, such as lesion features for most patients (e.g., drusen, subretinal fluid, LBD, and tumor thickness), were also obtained. In total, 18 510 UM images and 8671 nevus images were included in the study from a total of 3942 patients. An additional 1192 images of the opposite healthy eyes from 484 of the existing UM and nevus patients were also used for classification in this study. Training, test, and validation splits were taken at 70%, 20%, and 10%, respectively, randomized each time. Images of the same patients are used either in the training or testing to avoid information leakage. Image augmentation or enhancement was not performed on any of the image sets, to keep the results as close to “real world” as possible.

### Model

**Figure 2:**
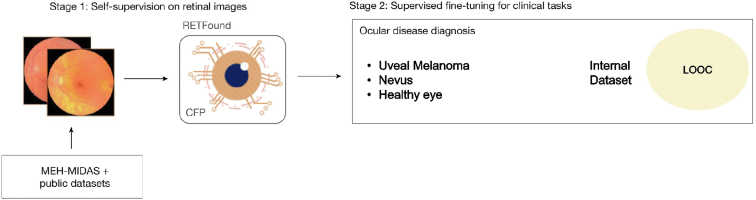
RETFound schematic for the 2-stage foundation model used in this study. The image was modified from the article by Zhou et al.^10^ CFP = color fundus photograph; LOOC = Liverpool Ocular Oncology Centre; MEH-MIDAS = Moorfields diabetic image dataset.

This study applied RETFound,^10^ a foundation self-supervised DL learning model, which was trained on >900 000 diabetic and public fundus images. The model can be fine-tuned for downstream classification tasks of multiple diseases, such as diabetes, heart disease and age-related macular degeneration. Figure 2 shows the RETFound architecture with stage 1, the self-supervised training, and stage 2, the fine-tuning of the model for disease classification. Using the UM, nevi, and healthy eye images obtained, RETFound was fine-tuned for binary and tertiary classification between UM and nevi as well as UM, nevi, and healthy control eyes. Images were resized to 224 × 224 and RETFound converts the images to gray scale. This allows for both color and auto fluorescent images to be used in this study.

### Hardware and Performance Metrics

Training, testing, and validation were performed on an NVIDIA GTX 4090 GPU with a batch size of 16 and a learning rate of 0.0005. For each of the results, a confusion matrix was plotted; for the binary classification, an area under the receiver operating characteristic curve (AUROC) was also plotted. Accuracy, sensitivity, specificity, F1-score, and Matthew’s correlation coefficient were calculated from the results. Matthew’s correlation coefficient was not calculated for the tertiary classification because it is more commonly used for binary confusion matrixes.

## Results

### Data Characteristics

Characteristics and clinical features for the remaining 4255 patients after preprocessing are shown in Table 1. The UM cohort had a slightly higher number of males whereas more females were present in the nevus group. Median age for the patient groups was not statistically significantly different (Mann–Whitney *U* test; *P* > 0.05). Uveal melanoma had a significantly greater LBD and tumor thickness when compared with nevi (Mann–Whitney *U* test; *P* > 0.05). Tumor stage was calculated using the tumor-node-metastasis/American Joint Committee on Cancer^13^ system, which uses the LBD and tumor thickness to categorize it into 1 of 4 stages. Although it is understood that the tumor-node-metastasis system is only applied in the clinic to malignancies, to allow for hypothetical size comparison between datasets, the tumor-node-metastasis system was also applied to the nevus cohort. In the UM group, the T3 stage was the most common, followed by T2, T1, and then T4. Most nevi were in the T1 group, with only a very few being classified as T2 and T3. As expected, there were no nevi in the T4 group. Drusen was present in approximately 50% of nevi but only in 8% of UM. Subretinal fluid showed the opposite relationship with its presence detected in 46% of UM but only 7% of nevi. Lipofuscin was present in 5% of nevus patients, with this parameter not being recorded in 29 patients.

**Table 1:** Demographic and Clinical Characteristics for All Patients Included in the Study (N = 4255)

Characteristics	Choroidal Melanoma (2073)	Nevi (1698)	Healthy (484)
Sex			
Male	1116	696	189
Female	957	1002	186
Age (yrs)			
Median (IQR)	62 (19–99)	66 (18–96)	60 (22–90)
Dimensions			
LBD (mm), median (IQR)	11.1 (1.4–24.1)	6.1 (0.5–18.0)	N/A
Tumor thickness (mm), median (IQR)	4.0 (0.2–18.3)	1.4 (0.1–9.9)	N/A
Tumor stage			
T1	626	1478	N/A
T2	633	202	N/A
T3	703	82	N/A
T4	108	0	N/A
No data	3	39	N/A
Drusen			
Yes	174	839	N/A
No	1856	830	N/A
No data	43	30	N/A
Subretinal fluid			
Yes	997	104	N/A
No	1033	1565	N/A
No data	43	30	N/A
Modality (number of images)			
Color fundus	12 476	5941	641
Autofluorescent	6034	2730	551

IQR = interquartile range; LBD = largest basal diameter; N/A = not applicable.

### Binary Classification

**Figure 3:**
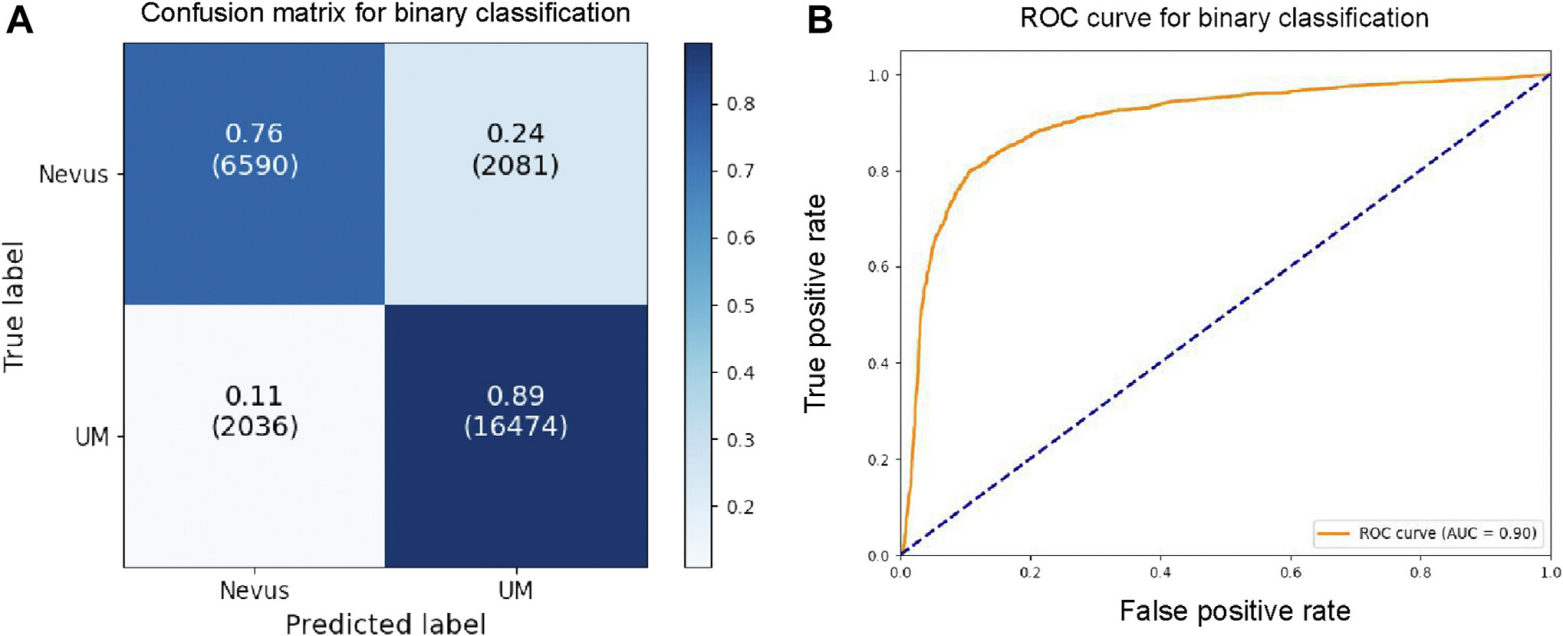
**A,** Confusion matrix for results of classification of fundus images of melanoma and nevi. **B,** Area under the receiver operating characteristic (ROC) plot of 0.90 for the model on the fundus images. AUC = area under the curve; UM = uveal melanoma.

The binary classification results of UM versus nevi are shown in Table 2 and Figure 3. Accuracy of the model was 0.83, demonstrating a good overall classification with a high specificity of 0.87 and sensitivity of 0.79. The F1 score and Matthew’s correlation coefficient also indicated strong model performance with 0.84 and 0.66 values, respectively. The receiver operating characteristic curve in Figure 3B has an AUROC value of 0.90 with the graph itself showing the curve reaching toward the top left-hand corner showing an excellent correct classification probability.

**Table 2:** Performance Metrics for Binary Classification Model

Metric	Value
Accuracy	0.83
Sensitivity	0.79
Specificity	0.87
F1 score	0.84
MCC	0.66
AUROC	0.90

AUROC = area under the receiver operating characteristic curve; MCC = Matthew’s correlation coefficient.

It was theorized that the model could struggle with the prediction of smaller tumors as they would be of a similar size to the average nevus. The model was therefore tested with T1 UM and all nevi, as well as the T2 UM and all nevi. This was also undertaken with the absence of drusen or subretinal fluid to determine if this had an impact on performance. T1 performance showed only a slight reduction in performance with an overall accuracy of 0.80, whereas T2 had an overall accuracy of 0.83. There was no significant change in model performance when assessed according to tumor stage. Comparing the model in cases without subretinal fluid and drusen increased the accuracy to 0.84 and 0.89, respectively.

### Tertiary Classification

**Figure 4:**
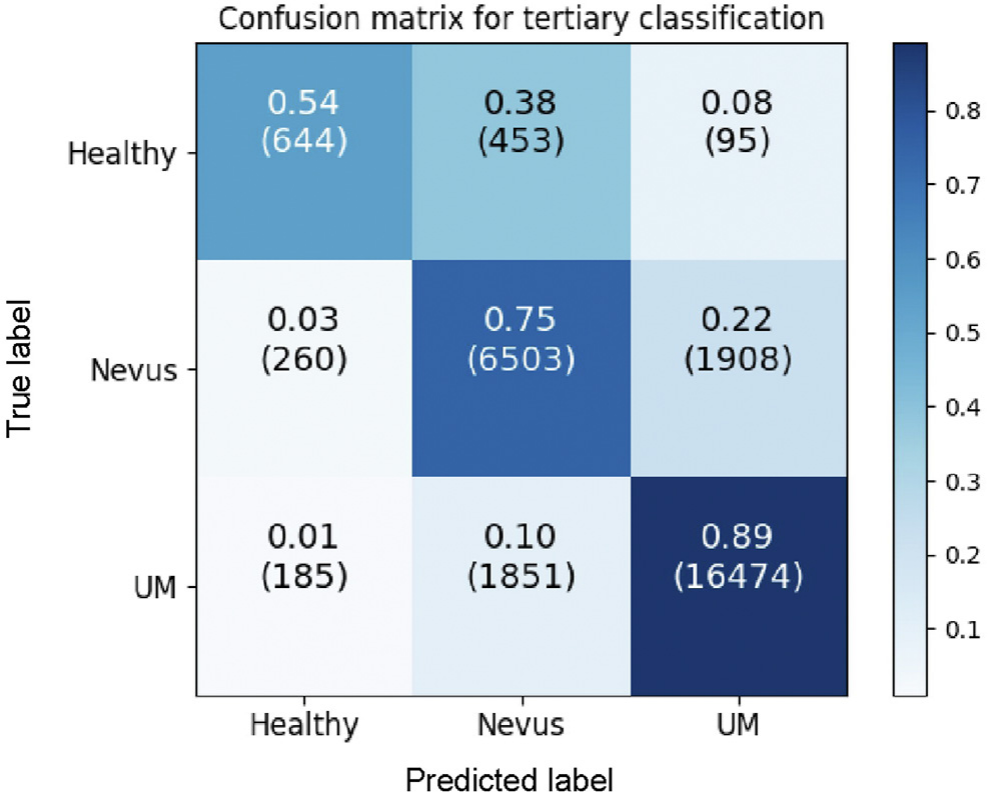
Confusion matrix for the results of classification of fundus images of melanoma, nevi, and healthy images. UM = uveal melanoma.

Figure 4 shows the confusion matrix for the tertiary classification of UM, nevus, and healthy eyes. The model showed an accuracy of 86% for UM with 76% for nevus and 83% for healthy eyes of the time. Performance values are shown in Table 2 with the weighted mean values taken for each, and Matthew’s correlation coefficient was not calculated for the reasons mentioned previously. The mean accuracy of the model was 82%, as shown in Table 3; however, because of the large dataset imbalance regarding case numbers, F1-score was a more adequate performance measurement. This had a value of 0.72, which showed a more accurate performance than what is shown in Figure 4. Mean specificity and sensitivity remained high with values of 85% and 73%, respectively. Similar to the binary classification, our DL model was more specific than sensitive as can also be seen in Table 3.

**Table 3:** Performance Metrics for 3 Class Confusion Matrix

Metric	Image	Value
Accuracy	Healthy	0.83
	Nevus	0.76
	UM	0.86
	**Mean**	**0.82**
Sensitivity	Healthy	0.54
	Nevus	0.75
	UM	0.89
	**Mean**	**0.73**
Specificity	Healthy	0.98
	Nevus	0.75
	UM	0.81
	**Mean**	**0.85**
F1 score	Healthy	0.68
	Nevus	0.67
	UM	0.81
	**Mean**	**0.72**
AUCROC	All	0.92

AUROC = area under the receiver operating characteristic curve; UM = uveal melanoma.

Bold indicates mean value.

## Discussion

Smaller UM and nevi share many overlapping clinical features posing a challenge for nonspecialized workers, such as opticians, to differentiate between the 2 lesions. This can lead to an increased number of unnecessary referrals to both general and specialized regional centers, increasing pressure on secondary and tertiary care services. The use of AI shows great promise in other areas of health care, and, therefore, testing the feasibility of its use on rare cancers such as UM is very important to enable earlier detection. In this novel study, a revised version of RETFound was fine-tuned and shows great promise for differentiating UM versus nevi. The use of a large, unedited dataset demonstrates a robust model with the potential for real-world applications.

The clinical information for the cases assessed in this study is shown in Table 1. It is known that males have a slightly increased risk of developing UM, which is consistent with our cohort.^14^ Nevi typically occur in men and women equally; however, in our dataset, the number of women was greater than men.^15^ People over the age of 50 years have a greater risk of developing UM, with the majority of the patients in our study being above this age and showing no statistically significant differences in median age between UM and nevi. The number of choroidal melanomas in this study was consistent with the literature, which reports approximately 85% to 90% of UM cases are located in the choroid.^16^ Largest basal diameter and tumor thickness showed statistically significant differences between the data sets agreeing that UM tends to be larger in size than nevi. Despite this, smaller T1 tumors are still detected with great accuracy and did not reduce the model’s performance allowing the model to be applied to any melanoma size. Drusen and subretinal fluid figures also agreed with current clinical practices with the presence of drusen indicating a nevus and subretinal fluid indicating a UM. Lipofuscin aids in the assessment of whether a choroidal melanocytic lesion is an unequivocal nevus or an indeterminant (borderline) lesion. Eight-eight cases with lipofuscin in our study were all confirmed nevi; any indeterminate or transformed lesions were excluded.

Our binary classification results show that more nevi are misdiagnosed as UM than UM as nevi. In this regard, it could be argued that false-positive cases cause less harm than having many false negatives, because of the implications of incorrect UM diagnosis on a patient’s treatment and prognosis. It should be noted that 11% of UM patients were misdiagnosed by the model as nevi. This could be due to the imbalance between image numbers in the UM and nevi datasets. Hence, by increasing the number of nevus images in future studies, we anticipate that the model’s performance will be enhanced leading to a decrease in the number of false positives in both categories. To make this model more feasible for routine clinical use, certain protocols would need to be set up to avoid these 11% of patients being misdiagnosed.

In the tertiary classification, the success rate of detecting UM and nevus was similar to the binary results; however, many of the healthy eyes were incorrectly classified and nevi were also most often misinterpreted by the model as healthy eyes. This is likely to be due to some nevi being very small or having less prominent features, causing the model to interpret these images as healthy eyes. In addition, the significantly lower number of healthy eyes compared with both UM and nevi could also cause this slight dip in performance. It again highlights the need to increase the numbers of both nevus and healthy eyes to be assessed by the model in future studies.

There are limited studies applying AI to ocular oncology images to date. Machine learning has been reported to be an effective method of differentiating between a small choroidal melanoma and a choroidal nevus by Zabor et al,^17^ producing an AUROC of 0.88. Unlike that model, our study did not use preidentified features or clinical input; instead, our model was allowed to extract its own features using its self-supervised learning backbone. More closely related to our study is the work of Cao et al^18^ who have shown the viability of DL on color fundus images, specifically using color fusion for enhanced accuracy. They reported a mean accuracy of 0.90 and AUROC of 0.93 and show the viability of AI to detect UM. However, their sample size was small: 157 UM and 281 nevi, with a total number of 798 images. Although Cao et al^18^ demonstrate good results, the smaller sample size could make the model prone to “overfitting,” and therefore is not as “true” to real-world imaging. Our model has greater accuracy for UM classification than theirs for nevus classification, which may relate to the larger number of UM images in our study, whilst they had more nevus images in theirs. Our model was also trained using both the color fundus and autofluorescence images together allowing for either image to be input and classified. It should be noted that both image types were not available for all patients, primarily because of the poor quality of images. Overall, both models had difficulty in classifying nevi from healthy eyes.

In summary, our study applying a fine-tuned RETFound for UM versus nevus classification demonstrates a good “proof-of-concept” (i.e., that it is possible to differentiate the 2 lesions using a self-supervised DL model as a backbone). The model can accurately predict UM but struggles slightly with the differentiation between nevi and healthy eyes, possibly because of the sample size imbalance. Our study also further emphasizes the capabilities of AI and, specifically, the use of foundation models on rare cancers, to provide good classification accuracy. Further work still needs to be undertaken to reduce the number of false-positive results and to increase the accuracy of nevi and healthy eye classification. To this end, external validation sets are being acquired in the context of a multicenter study, which will commence in the near future. We are confident that the model’s excellent performance will hold-up on further analysis of its ability for automated UM/nevus differentiation.
